# Domain‐Targeted RNAi of VeA Reveals Its Essential Role in the *Fusarium oxysporum*–*Pseudostellaria heterophylla* Interaction

**DOI:** 10.1111/mpp.70257

**Published:** 2026-04-24

**Authors:** Zhen Fang, Ludi Yang, Yuping Li, Jianing Guo, Quancheng Sun, Shengen Zhong, Yanyang Jiao, Chenjing Zhang, Wenxiong Lin

**Affiliations:** ^1^ College of Life Sciences Fujian Agriculture and Forestry University Fuzhou Fujian Province China; ^2^ Fujian Key Laboratory of Agroecological Processing and Safety Monitoring, College of Agriculture Fujian Agriculture and Forestry University Fuzhou China; ^3^ Key Laboratory of Crop Ecology and Molecular Physiology (Fujian Agriculture and Forestry University), College of Agriculture Fujian Province University Fuzhou China; ^4^ College of Agriculture Fujian Agricultural and Forestry University Fuzhou China

**Keywords:** *F. oxysporum* f. sp. *pseudostellariae*, pathogenicity, *Pseudostellaria heterophylla*, RNAi, VeA

## Abstract

*Fusarium oxysporum* f. sp. *pseudostellariae* (Fop) is the major causal agent of root rot in *Pseudostellaria heterophylla*. This study focused on VeA, a conserved pathogenicity regulator in Fop. RNA interference constructs targeting the Velvet domain (VeA‐1) and nuclear localization signal (NLS) region (VeA‐2) were generated to assess their effects on fungal growth, virulence and host responses. Phylogenetic and structural analyses showed that Fop VeA contains a canonical Velvet domain and NLS motif conserved across filamentous fungi. Silencing of *VeA* significantly impaired colony growth and conidiation, indicating its positive regulatory role in development. Pathogenicity assays revealed markedly reduced virulence of ΔVeA‐1 and ΔVeA‐2 strains, with alleviated root rot symptoms in *P. heterophylla* . Biochemical assays showed that wild‐type infection induced strong antioxidant enzyme activities (peroxidase, catalase, superoxide dismutase), whereas these responses were attenuated under *VeA* silencing, corresponding to reduced oxidative stress. Reverse transcription‐quantitative PCR confirmed that *VeA* modulates host defence gene expression (*PR2*, *PR4*, *PR5*). Transcriptomic enrichment further indicated activation of immune‐related pathways during infection. Collectively, VeA acts as a key regulator coordinating fungal development and virulence while indirectly manipulating host oxidative and immune responses, providing a potential molecular target for biological control of root rot in *P. heterophylla* .

## Graphical abstract

*Fusarium oxysporum* f. sp. *pseudostellariae* VeA, containing a conserved Velvet domain and nuclear localization signal (NLS), regulates growth, conidiation and virulence. *VeA* silencing weakened pathogenicity, reduced reactive oxygen species accumulation and activated host defence, suggesting VeA is a key virulence regulator and potential target for SIGS‐based control.

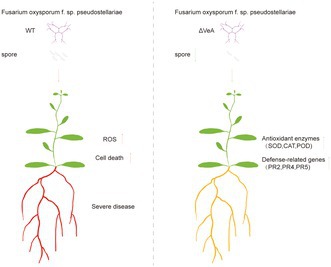

## 1 Introduction

*Fusarium oxysporum* is a globally distributed and highly destructive soil‐borne pathogen characterized by strong host specialization (forma specialis) and its ability to cause vascular wilt diseases; it results in severe economic losses in numerous crops, including tomato, cotton, banana, tobacco and various medicinal plants. Typical symptoms include vascular bundle browning and xylem necrosis caused by fungal colonization and occlusion of vascular tissues, progressive wilting and eventual plant death. Its strong persistence in soil, combined with the limited efficacy of chemical control measures and the shortage of resistant cultivars, makes it a major challenge in global agriculture. Increasing evidence indicates that the enhanced pathogenicity of *F. oxysporum* stems from its unique ‘mobile pathogenicity chromosomes’, which drive host adaptation and virulence expansion (Ma et al. 2010; Ma 2014; van Dam et al. 2017).

During fungal pathogenesis, environmental sensing, developmental transitions and secondary metabolism are tightly connected processes. Recent studies have established that the regulatory module composed of Velvet‐family proteins (e.g., VeA, VelB, VosA) and the epigenetic regulator LaeA functions as a central hub controlling fungal development and virulence, particularly in phytopathogenic species. This module coordinates asexual sporulation, aerial mycelial formation and the expression of toxins and effector proteins (Yu et al. 2023; Hou et al. 2024). VeA plays a crucial role as a transcriptional regulator whose nuclear localization, dimerization with VelB and interaction with LaeA collectively affect reproductive mode transitions and virulence factor activation (Hou et al. 2024). Across diverse phytopathogens, deletion of VeA or LaeA typically leads to reduced virulence, impaired conidiation, disrupted cell wall integrity and decreased secondary metabolism, underscoring their conserved roles as pathogenicity regulators (Wu et al. 2024; Calvo et al. 2024). Nonetheless, the Velvet–LaeA module exhibits species‐specific regulatory differences within *Fusarium*, suggesting a key involvement in host specialization and virulence evolution (Yu et al. 2023).

*Fusarium oxysporum* f. sp. *pseudostellariae* (Fop), a specialized form infecting *Pseudostellaria heterophylla*, severely restricts the sustainable development of this medicinal crop. Fop infection causes root black rot, vascular necrosis and yield decline, and is closely associated with continuous cropping obstacles (Zhao et al. 2015; Yuan et al. 2022; Qin et al. 2017). Although effector proteins and toxins of *F. oxysporum* have been widely reported, upstream molecular regulators of pathogenicity in Fop remain largely unexplored. In particular, the roles of the Velvet regulatory module in mediating developmental regulation and virulence in this specialized pathogen have not been systematically investigated.

RNA interference (RNAi), a conserved eukaryotic gene‐silencing mechanism, participates in stress adaptation, gene regulation and fungal virulence as well as host–pathogen interactions (Nakayashiki 2005; Leng et al. 2011; Lax et al. 2020; Liu et al. 2024). Recent advances in RNAi‐based applications have enabled functional characterization and disease management innovations, including non‐transgenic strategies such as spray‐induced gene silencing (SIGS) and nano‐ or vesicle‐mediated delivery systems (Koch et al. 2016; Waqas Choudry et al. 2024; Zhao and Guo 2022). Moreover, the discovery of cross‐kingdom small RNA (sRNA) trafficking provides a mechanistic basis for bidirectional gene regulation between plants and pathogens (Cai et al. 2018).

Although the Velvet module is known to be a core regulator of fungal development and metabolism, its contribution to Fop pathogenicity and its linkage to host oxidative stress responses remain unclear. In this study, targeted RNAi silencing of the *VeA* gene in Fop was performed. Through integrated analysis of fungal phenotypes, pathogenicity and transcriptomic regulation, we try to elucidate the role of VeA in Fop development, virulence and manipulation of host immunity, and evaluate its potential as a biocontrol target, including the prospect of SIGS‐mediated disease control.

## 2 Results

### 2.1 Amino Acid Sequence Analysis of the *VeA* Gene From Fop

**FIGURE 1:**
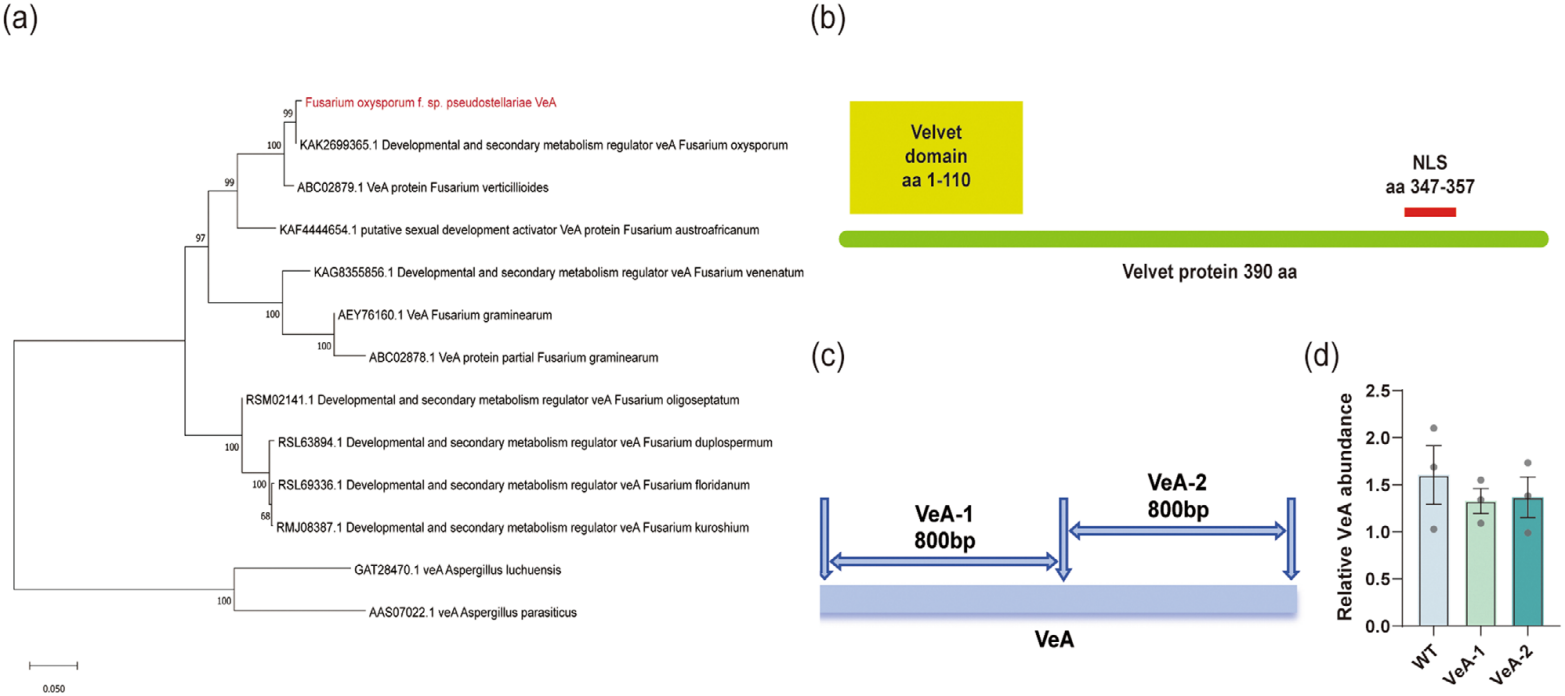
Sequence and structural analysis of the *VeA* gene in *Fusarium oxysporum* f. sp. *pseudostellariae*. (a) Phylogenetic analysis of VeA proteins. A neighbour‐joining phylogenetic tree was constructed to illustrate the evolutionary relationships of VeA proteins from various *Fusarium* species (e.g., *F. oxysporum*, *F. graminearum*, *F. verticillioides*) and *Aspergillus* species (e.g., *A. luchuensis*, *A. parasiticus* ). Bootstrap support values (based on 1000 replicates) are indicated on the branches. (b) Schematic of the Velvet protein domain architecture. The Velvet protein consists of 413 amino acids (aa). The conserved Velvet domain spans aa 1–110, and a nuclear localization signal (NLS) is located at aa 347–357. (c) Schematic representation of the PCR amplification strategy for the *VeA* gene. Two primer pairs, VeA‐1 and VeA‐2, were designed to amplify ~800 bp fragments of the *VeA* gene. (d) Relative genomic *VeA* copy number was quantified by quantitative PCR in the wild type (WT) and two independent VeA‐RNAi transformants (VeA‐1 and VeA‐2). Bars represent mean ± SD (*n* = 3), with individual data points shown as dots.

To investigate how *P. heterophylla* responds to RNAi‐silenced strains of Fop, we first conducted a phylogenetic analysis of the cloned *VeA* (Figure 1a). The results showed that the encoded VeA protein belongs to the canonical Velvet regulatory family in filamentous fungi and exhibits extremely high conservation with VeA homologues from *Fusarium verticillioides*, *Fusarium graminearum* and several *Aspergillus* species, supported by robust bootstrap values of 99%. A multiple sequence alignment of Fop VeA with its homologues further confirmed this high conservation (Figure S1). These findings align with previous reports emphasizing the evolutionary conservation of *VeA* in *Fusarium* spp. (Bayram and Braus 2012), suggesting that VeA acts as a stable core regulator of fungal development and virulence. This provides a strong evolutionary basis for further functional characterization of VeA in the Fop– *P. heterophylla* interaction.

Domain structure analysis (Figure 1b) revealed a typical N‐terminal Velvet domain (1–110 amino acids [aa]) and a C‐terminal nuclear localization signal (NLS; 347–357 aa) within Fop VeA. The Velvet domain is critical for protein–protein interactions and the formation of regulatory complexes that cooperate with the epigenetic regulator LaeA to modulate downstream gene expression and secondary metabolism (Bayram et al. 2008). The presence of an NLS indicates that Fop VeA primarily functions within the nucleus. Similar structural features in *Aspergillus nidulans* have been shown to govern developmental differentiation and toxin synthesis (Kato et al. 2003). Deep bioinformatic analysis of Fop VeA's conserved domains and NLS (including SMART, InterPro, DeepLoc2.0 and cNLS Mapper predictions) further validated these structural characteristics (Figure S2). These conserved characteristics imply that Fop VeA likely forms regulatory complexes following nuclear translocation to modulate fungal growth, development and pathogenicity toward *P. heterophylla* . More bioinformatic analyses are in Figure S2.

Guided by these structural insights, a segmental RNAi strategy was devised to functionally dissect VeA (Figure 1c). RNAi is a powerful tool for fungal gene functional analysis and virulence studies (Trieu et al. 2017). We amplified two approximately 800 bp fragments corresponding to the Velvet domain (VeA‐1) and NLS‐containing region (VeA‐2), and used them to generate two *VeA*‐silenced strains via protoplast transformation. All primers used in this study are listed in Table S1. These mutants were subsequently employed to investigate how silencing distinct VeA functional regions differentially affects Fop pathogenicity and host defence activation.

Also, we compared the relative copy number of the *VeA* gene in the wild‐type strain (WT) and two VeA‐RNAi transformants (VeA‐1 and VeA‐2) (Figure 1d). Quantitative PCR (qPCR) analysis showed that there were no significant differences in the relative *VeA* copy number between WT and the two RNAi strains. Although some variation was observed between the transformants, their overall *VeA* copy numbers tended to be slightly lower than that of WT. The standard curves for qPCR of Fo‐*Tub* (reference) and Fo‐*VeA* quantification exhibited excellent linearity, confirming the reliability of the assay (Figure S3). These results indicate that VeA‐RNAi transformation did not introduce additional copies of the *VeA* gene, and that *VeA* remains a low‐copy gene in the transformants, thereby excluding the possibility that the observed phenotypic changes were caused by *VeA* copy number amplification.

### 2.2 *VeA* Silencing Disrupts Growth and Reproductive Development in Fop

**FIGURE 2:**
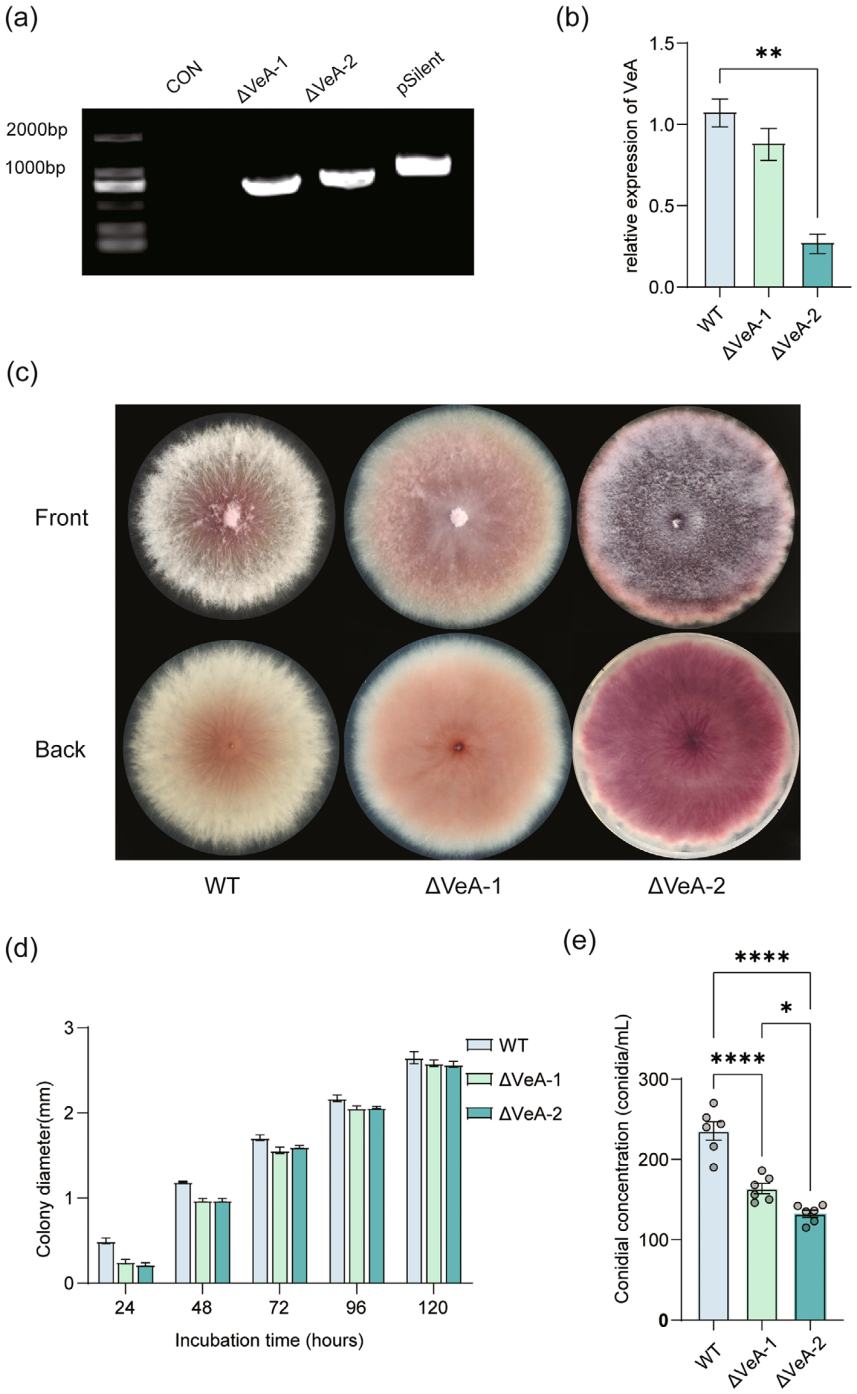
Molecular identification of *VeA*‐silenced strains of *Fusarium oxysporum* f. sp. *pseudostellariae* (Fop) and the effects of *VeA* silencing on colony growth and spore formation of Fop. (a) PCR amplification used to verify *VeA* gene silencing in wild type (WT), two *VeA*‐silenced strains (ΔVeA‐1 and ΔVeA‐2) and the pSilent empty vector control (lanes arranged in the indicated order). (b) Relative expression levels of *VeA* in WT, ΔVeA‐1 and ΔVeA‐2. (c) Colony morphology of WT and *VeA*‐silenced strains. Upper and lower panels represent the front and reverse sides of the colonies, respectively. (d) Growth curves showing colony diameter of WT, ΔVeA‐1 and ΔVeA‐2 measured at 24, 48, 72, 96 and 120 h post‐incubation. (e) Comparison of conidial concentration (conidia mL^−1^) among WT, ΔVeA‐1 and ΔVeA‐2. All data are presented as mean ± SD (*n* = 6) using a one‐way ANOVA followed by Duncan's multiple range test. Asterisks indicate significant differences (*p < 0.05, **p < 0.01, ****p < 0.0001).

To investigate the role of the *VeA* gene in the growth and development of Fop, a *VeA*‐targeted RNAi silencing construct based on the pSilent system was developed and introduced into Fop protoplasts. Two distinct silenced strains, ΔVeA‐1 and ΔVeA‐2, were successfully obtained. PCR validation of the pSilent recombinant vectors and *VeA* target fragment amplification confirmed the successful construction of the silencing system (Figure S4). PCR detection of the hygromycin resistance marker (~1000 bp) carried by the vector revealed clear amplification in both mutants (Figure 2a), confirming stable genomic integration of the silencing cassette. Moreover, reverse transcription (RT)‐qPCR analysis demonstrated a significant reduction in *VeA* transcript abundance in both strains (Figure 2b), with ΔVeA‐2 displaying the highest silencing efficiency (*p* < 0.01). These results indicate that the RNAi system effectively suppresses *VeA* transcription and that silencing efficiency varies across different targeted regions, likely attributable to the essential role of the nuclear localization signal (NLS) in VeA's nuclear function and transcriptional regulation (Stinnett et al. 2007; Bayram and Braus 2012).

Morphologically, the silenced strains exhibited pronounced growth alterations compared with the wild type (WT) (Figure 2c). WT colonies showed abundant aerial hyphae and a typical fluffy appearance, whereas ΔVeA‐1 and ΔVeA‐2 developed flatter, denser colonies with enhanced pigment accumulation. Growth curve analysis further revealed reduced colony diameters in both mutants at multiple time points (Figure 2d). These phenotypes align well with the conserved role of Velvet proteins in controlling vegetative growth (Bayram et al. 2008; López‐Berges et al. 2014).

Silencing of *VeA* also impaired sporulation. Both mutants exhibited markedly reduced conidial production relative to WT (Figure 2e), with the most drastic decrease observed in ΔVeA‐2 (*p < 0.05; ****p < 0.0001). These findings indicate that VeA is required for efficient asexual reproduction and that its disruption compromises both fungal fitness and its potential for spread and infection.

Meanwhile, we examined the relative transcriptional levels of Argonaute, Dicer1‐like and Dicer2‐like genes in Fop wild‐type (WT) strain and two *VeA* deletion mutants (ΔVeA‐1 and ΔVeA‐2). These three genes encode canonical components of the RNAi pathway. As shown in Figure S5, compared with the WT, all three genes exhibited an upward trend in expression in both ΔVeA‐1 and ΔVeA‐2, indicating that the pSilent system was successfully introduced into Fop and is functionally active. The recombinantly expressed double‐stranded RNA (dsRNA) fragment is processed by Dicer‐like proteins into small interfering RNAs (siRNAs), which subsequently associate with Argonaute to target complementary mRNAs for degradation, thereby achieving gene silencing. Notably, the upregulation of these RNAi pathway genes was more pronounced in ΔVeA‐2, indirectly suggesting that this mutant may possess higher RNA silencing efficiency.

Overall, VeA functions as a positive regulator of vegetative growth, pigmentation and sporulation in Fop and exhibits regulatory characteristics typical of Velvet family proteins. Additionally, targeting the NLS region yielded a more efficient silencing effect, supporting its critical functional role in VeA‐mediated regulation.

### 2.3 VeA As a Central Regulator Linking Fungal Virulence and Host Development in the Fop–*P. heterophylla* Pathosystem

**FIGURE 3:**
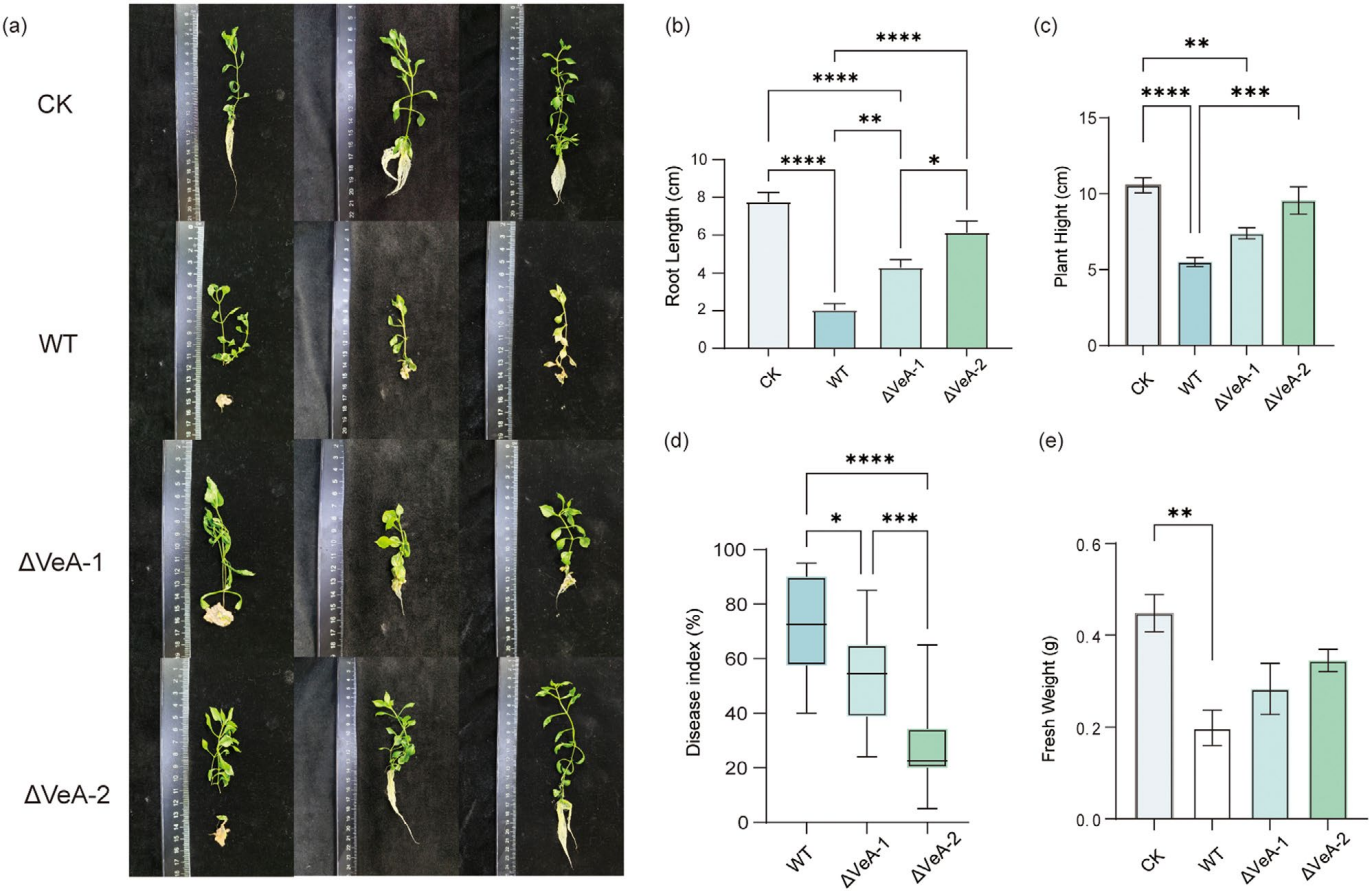
*VeA* silencing reduces the pathogenicity of *Fusarium oxysporum* f. sp. *pseudostellariae* (Fop) toward *Pseudostellaria heterophylla*. (a) Representative phenotypes of plants inoculated with mock control (CK), wild type (WT) and two independent ΔVeA mutants (ΔVeA‐1 and ΔVeA‐2). (b) Root length, (c) plant height, (d) disease index and (e) fresh weight were measured. Data are presented as mean ± SD (*n* = 10) using a one‐way ANOVA followed by Duncan's multiple range test. Asterisks indicate significant differences (*p < 0.05, **p < 0.01, ***p < 0.001, ****p < 0.0001).

Phenotypic evaluation following rhizosphere inoculation (Figure 3a) clearly revealed differences in virulence among the tested strains in *P. heterophylla* . Mock‐inoculated plants (CK) exhibited healthy growth, characterized by robust primary roots, abundant lateral roots, intact leaves and absence of visible symptoms. In contrast, plants inoculated with the WT strain developed severe root atrophy accompanied by browning and rot, along with pronounced leaf wilting, indicating the strong virulence of Fop. Notably, plants inoculated with the ΔVeA‐1 and ΔVeA‐2 strains displayed markedly improved root morphology and substantially alleviated disease symptoms, suggesting that silencing of *VeA* significantly mitigates Fop‐induced tissue damage.

Quantitative analyses of root length and plant height further substantiated these observations (Figure 3b,c). The CK group exhibited the longest roots (approximately 7.75 cm), whereas root elongation was severely inhibited in WT‐inoculated plants, reaching only about 2 cm. In contrast, ΔVeA‐1 and ΔVeA‐2 restored root length to approximately 4.3 and 6 cm. Similarly, plant height in the CK group averaged approximately 10 cm, while WT‐infected plants were reduced to approximately 5.5 cm. Inoculation with ΔVeA‐1 and ΔVeA‐2 partially restored plant height to approximately 7.5 and 9.4 cm, respectively. These results indicate that loss of VeA markedly impairs the pathogen's capacity to disrupt root development and suppress host growth.

Assessment of disease severity provided direct evidence for reduced virulence (Figure 3d). WT‐inoculated plants exhibited a disease index of approximately 75%, whereas the disease index decreased to approximately 55% in ΔVeA‐1 and further declined to approximately 23% in ΔVeA‐2. Consistently, fresh weight measurements demonstrated partial recovery of plant biomass in ΔVeA‐inoculated plants compared with WT (Figure 3e). These trends closely paralleled changes in sporulation capacity, supporting the notion that VeA coordinately regulates Fop reproductive fitness and infection efficiency.

Collectively, these findings identify VeA as a critical determinant of Fop pathogenicity. Normal expression of VeA enables effective host colonization, root tissue destruction and growth suppression, whereas its silencing leads to a pronounced attenuation of virulence. By integrating phenotypic traits, biomass‐related parameters and disease progression indices, this study highlights the central role of VeA in driving pathogenic development during the Fop– *P. heterophylla* interaction.

### 2.4 Physiological Defence Responses of *P. heterophylla* Under Different Treatment Conditions

During plant–pathogen interactions, the rapid accumulation of reactive oxygen species (ROS) represents one of the earliest host defence responses to pathogen invasion. Although ROS are indispensable for signal transduction and antimicrobial activity, excessive accumulation can lead to oxidative toxicity and cellular damage. Therefore, the antioxidant enzyme system—centred on peroxidase (POD), catalase (CAT) and superoxide dismutase (SOD)—plays a critical role in maintaining host redox homeostasis (Sharma et al. 2012; Huang et al. 2019). To elucidate how VeA of Fop modulates the antioxidant response and growth status of *P. heterophylla* , we systematically analysed relevant physiological and biochemical parameters.

**FIGURE 4:**
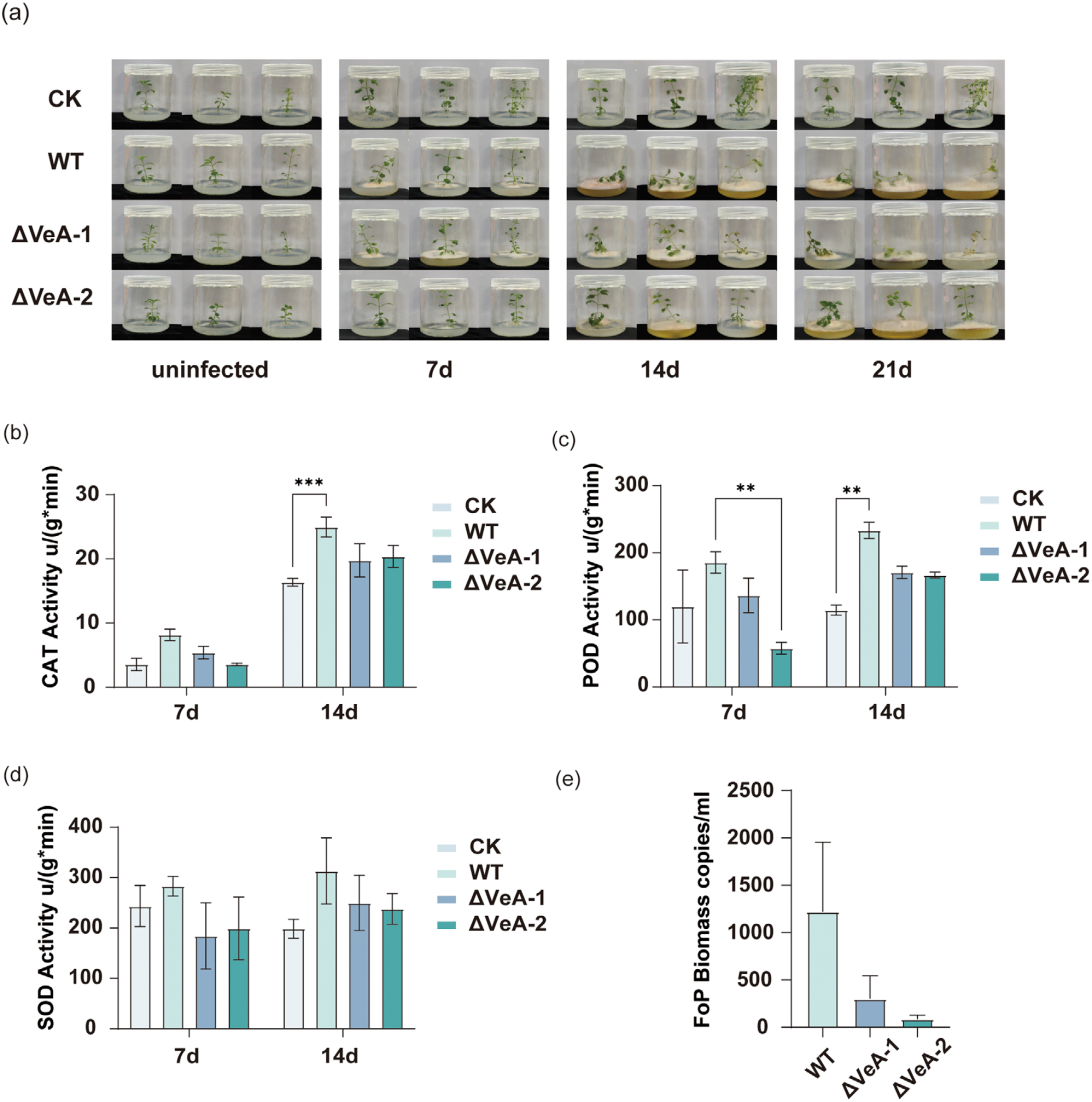
Changes in physiological defence responses of *Pseudostellaria heterophylla* tissue culture seedlings. (a) Growth phenotypes of *P. heterophylla* plants inoculated with wild type (WT) *Fusarium oxysporum* f. sp. *pseudostellariae* (Fop) or *VeA*‐silenced mutants (ΔVeA‐1 and ΔVeA‐2) at 0, 7, 14 and 21 days post‐inoculation (dpi). Mock‐inoculated plants (CK) served as the control. (b–d) Antioxidant enzyme activities in roots at 7 and 14 dpi, (b) catalase (CAT), (c) peroxidase (POD) and (d) superoxide dismutase (SOD). (e) Relative Fop biomass in the rhizosphere at 14 dpi determined by quantitative PCR. Data are presented as mean ± SD (*n* = 6). Asterisks indicate significant differences (**p < 0.01, ***p < 0.001; two‐way ANOVA followed by post hoc tests).

As shown in Figure 4a, mock‐inoculated plants (CK) maintained healthy growth throughout the 21‐day observation period. In contrast, inoculation with the wild‐type (WT) Fop strain resulted in severe growth inhibition. By Day 14, plants exhibited pronounced wilting and tissue browning, accompanied by a noticeable discoloration of the growth medium, indicative of extensive pathogen colonization and metabolite accumulation. By Day 21, WT‐inoculated plants were nearly completely collapsed. In comparison, plants inoculated with the *VeA*‐silenced mutants (ΔVeA‐1 and ΔVeA‐2) displayed markedly improved growth performance, with substantially alleviated stress symptoms and relatively normal development throughout the experimental period.

Biochemical analyses further substantiated these phenotypic differences. At Day 7, CAT activity remained at relatively low levels across all treatments. By Day 14, however, CAT activity in WT‐inoculated plants increased sharply and was significantly higher than that in CK and ΔVeA groups, reflecting an urgent requirement for H_2_O_2_ detoxification under severe pathogenic stress (Figure 4b). Although CAT activity in ΔVeA‐1 and ΔVeA‐2 plants showed a moderate increase, it remained significantly lower than in WT‐infected plants, indicating that *VeA* silencing effectively alleviated H_2_O_2_‐mediated oxidative stress.

A similar trend was observed for POD activity (Figure 4c). At Day 7, POD activity in the WT group was significantly higher than in CK and ΔVeA groups. By Day 14, POD activity further increased in WT‐infected plants, whereas ΔVeA groups exhibited intermediate levels—higher than CK but significantly lower than WT—suggesting reduced oxidative pressure while maintaining a certain degree of defence activation. SOD activity displayed a comparable pattern (Figure 4d), with significantly elevated levels in WT‐treated plants at both Day 7 and Day 14, whereas no significant differences were observed between ΔVeA and CK groups. These results are consistent with an overall alleviation of oxidative stress following *VeA* silencing. Notably, physiological and biochemical parameters were not assessed at Day 21 because pathogen‐inoculated plants had already undergone severe deterioration, rendering such measurements unreliable.

In addition, quantitative analysis of Fop biomass (Figure 4e) revealed that the WT strain accumulated significantly higher copy numbers in the host rhizosphere compared with ΔVeA‐1 and ΔVeA‐2, directly confirming that loss of VeA markedly impairs pathogen colonization and proliferation. The standard curves for qPCR of Fop biomass quantification exhibited excellent linearity, confirming the reliability of the assay (Figure S3).

Taken together, these findings demonstrate that VeA acts as a positive regulator of Fop virulence. By promoting pathogen colonization, VeA enhances ROS overproduction in the host, thereby triggering a strong antioxidant response that ultimately suppresses plant growth. In contrast, *VeA* silencing effectively restricts pathogen proliferation and mitigates oxidative damage, thereby improving the physiological status and disease tolerance of *P. heterophylla* .

### 2.5 Comparative Expression Analysis of Defence‐Related Genes in *P. heterophylla* Responding to Different Fop Infection Treatments

**FIGURE 5:**
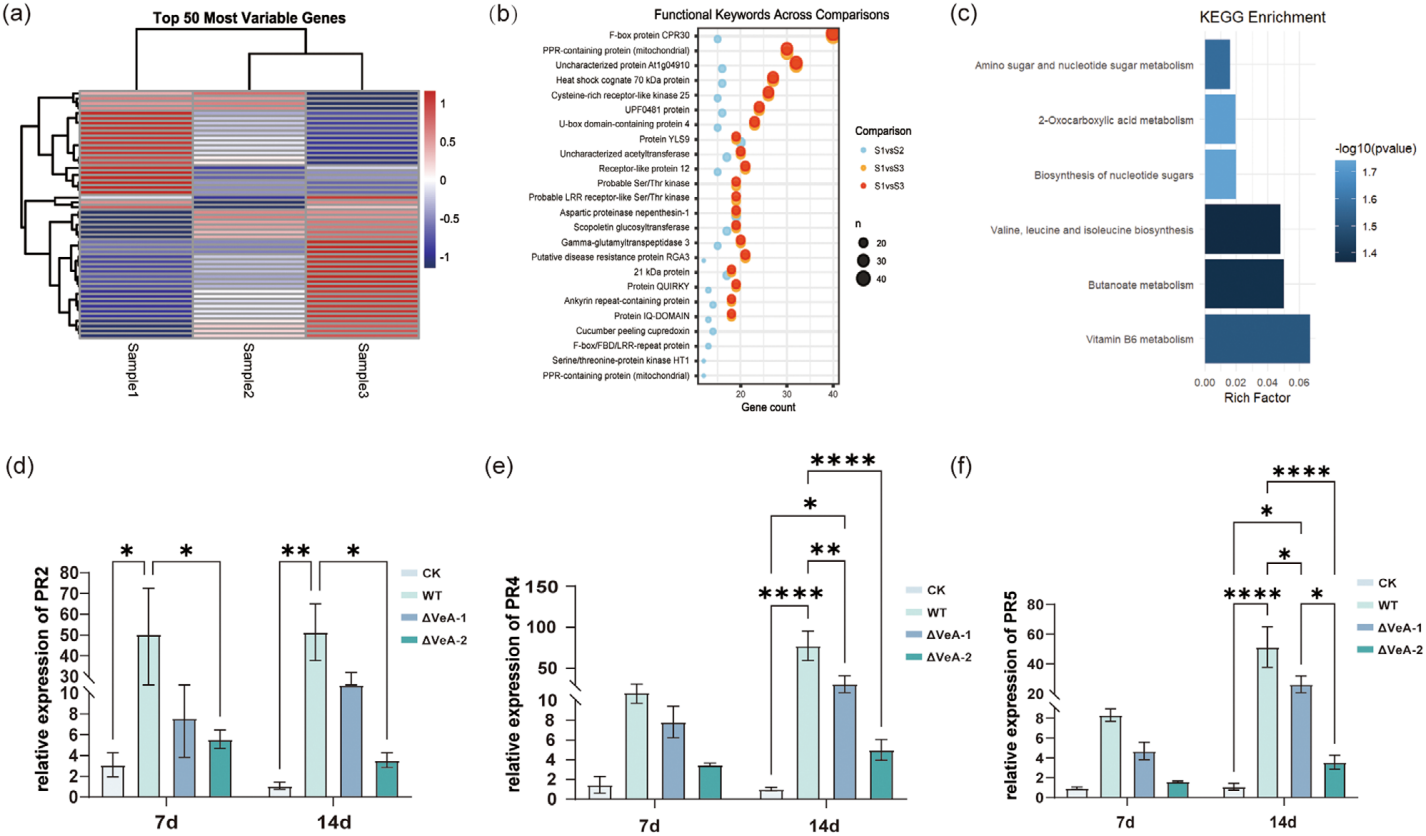
Analysis of the expression patterns of disease resistance‐related genes in *Pseudostellaria heterophylla*. (a) Heatmap and hierarchical clustering analysis of the top 50 most variable genes, illustrating the differential expression patterns of genes in Sample 1 (S1; uninfected control), Sample 2 (S2; infected with *Fusarium oxysporum* f. sp. *pseudostellariae* [Fop], 6 days post‐inoculation [dpi]) and Sample 3 (S3; infected with Fop, 13 dpi). The colour gradient denotes the normalized expression level of genes (red for high expression, blue for low expression) and the dendrogram reflects the similarity of expression patterns among genes and samples. (b) Bubble plot of gene enrichment for functional keywords across different comparisons (S1 vs. S2, S1 vs. S3, S2 vs. S3). The *x*‐axis represents the gene count, the *y‐*axis represents functional keywords, bubble colours distinguish comparison groups and bubble sizes represent the number of related genes (*n*). (c) Bar plot of KEGG pathway enrichment analysis. The *x*‐axis ‘Rich Factor’ is the ratio of the number of enriched genes in a pathway to the number of background genes. The *y*‐axis represents enriched metabolic pathways. The colour gradient corresponds to (−log(*p*value))(the larger the value, the higher the enrichment significance), showing significant enrichment of pathways such as 'Amino sugar and nucleotide sugar metabolism', and 'Valine, leucine and isoleucine biosynthesis'. (d–f) Relative expression analysis of pathogenesis‐related genes (PR genes) in *P. heterophylla* . The transcription levels of *PR2* (β‐1,3‐glucanase), *PR4* (chitinase) and *PR5* (thaumatin‐like protein) were measured by reverse transcription‐quantitative PCR in the control (CK), wild‐type strain (WT) inoculated and *VeA*‐silenced strains (ΔVeA‐1, ΔVeA‐2) inoculated plants at 7 and 14 dpi. Expression levels were normalized to the internal reference gene *β‐actin*. Data are presented as mean ± SD (*n* = 3 biological replicates × 3 technical replicates). Asterisks indicate significant differences (*p < 0.05, **p < 0.01, ****p < 0.0001; two‐way ANOVA followed by post hoc tests).

Upon pathogen invasion, plants undergo rapid transcriptome reprogramming to activate multilayered immune networks (Dodds and Rathjen 2010; Tsuda and Somssich 2015). To investigate the effects of Fop infection on the gene expression regulation and metabolic responses of *P. heterophylla* , three treatment groups were established: an uninfected control (Sample 1), a 6 days post‐inoculation (dpi) group (Sample 2) and a 13 dpi group (Sample 3). Based on RNA‐seq data and subsequent bioinformatics analyses, a series of core figures (Figure 5a–f) were generated to illustrate the molecular responses at different infection stages—from global transcriptional profiles and functional enrichment to key gene validation.

We present the heatmap and hierarchical clustering analysis of the top 50 most variable genes, revealing distinct expression profiles among the three groups (Figure 5a). The uninfected Sample 1 is clearly separated from the infected groups (Sample 2 and Sample 3), as shown by the contrasting colour gradients (red indicating high expression, blue indicating low expression). Sample 2 (6 dpi) and Sample 3 (13 dpi) display more similar expression patterns, consistent with the clustering dendrogram where these two groups first cluster together before merging with Sample 1. This pattern suggests that the overall transcriptional profile progressively diverges from the uninfected state following infection, and that the late‐stage (13 dpi) expression pattern is more closely aligned with the mid‐stage (6 dpi). These highly variable genes likely represent core response targets and provide a foundation for subsequent functional analysis.

Figure 5b displays a bubble plot of functional keyword enrichment across the three pairwise comparisons. The bubbles corresponding to Sample 1 vs. Sample 3 are generally larger and contain more genes, enriched in essential functions such as ‘stress response’, ‘defence response’, and ‘metabolic regulation’, indicating pronounced functional shifts under prolonged infection. In contrast, Sample 1 vs. Sample 2 (uninfected vs. 6 dpi) is mainly enriched for ‘signal transduction’ and ‘substance transport’ processes, reflecting the rapid activation of early defence signalling. The relatively fewer enriched terms in Sample 2 vs. Sample 3 imply that the transition from mid‐ to late‐stage infection involves more focused, stabilized metabolic and regulatory adjustments.

Figure 5c shows the KEGG pathway enrichment results, identifying the major metabolic pathways involved in the Fop infection response. The *x‐*axis (‘Rich Factor’) represents the ratio of enriched genes within each pathway to the total background genes, while the colour gradient reflects the −log_10_(*p*‐value), where higher values indicate stronger enrichment significance. Notably, ‘Amino sugar and nucleotide sugar metabolism’ and ‘Valine, leucine and isoleucine biosynthesis’ pathways were significantly enriched. The former participates in the synthesis and modification of cell wall components (e.g., chitin, glucan), suggesting a potential reinforcement of cell wall defences against Fop invasion. The latter pathway, crucial for energy metabolism and stress signalling, implies that *P. heterophylla* sustains physiological homeostasis by modulating amino acid metabolism under pathogen‐induced stress.

Building upon these findings, we further examined the expression patterns of three key pathogenesis‐related (PR) genes—*PR2*, *PR4* and *PR5* (Figure 5d–f). At both 7 and 14 dpi, the transcript levels of *PR2*, *PR4* and *PR5* in the WT‐inoculated group were significantly higher than those in the mock control (CK), indicating that Fop infection triggers a pronounced defence response in *P. heterophylla* . Notably, in plants inoculated with the ΔVeA‐1 and ΔVeA‐2 mutants, the expression of *PR2* (β‐1,3‐glucanase) at 14 dpi was significantly higher than that in the WT group (*p* < 0.05). Moreover, the transcript levels of *PR4* (chitinase‐like protein) and *PR5* (thaumatin‐like protein) were significantly elevated in the ΔVeA groups compared with WT at both 7 and 14 dpi (*p* < 0.05 or *p* < 0.0001). Overall, PR gene expression in ΔVeA‐infected plants was comparable to or even higher than the basal defence level observed in CK plants. These results suggest that loss of VeA leads to enhanced activation of host defence‐related genes, thereby strengthening resistance against Fop infection.

In summary, the integrated transcriptomic and validation results comprehensively depict the molecular response of *P. heterophylla* to Fop infection. The infection causes a clear shift in gene expression profiles between uninfected and infected samples, with progressively focused enrichment in defence‐ and stress‐related pathways as infection advances from 6 to 13 dpi. The specific activation of PR family genes and the regulatory role of VeA together constitute the core of the host defence network. In the ΔVeA treatment group, the metabolic response of *P. heterophylla* was markedly reduced, indicating that silencing the *VeA* gene significantly decreased its pathogenicity toward *P. heterophylla* .

### 2.6 Mechanism by Which VeA Regulates Fop Pathogenicity and Triggers Antioxidant and Defence Responses in *P. heterophylla*

In the field of medicinal plant disease control and molecular interaction research, Fop—a lethal pathogen causing root rot in *P. heterophylla* —holds significant industrial and scientific importance for elucidating pathogenic mechanisms and developing disease resistance strategies. This study focuses on the functional domains of the *VeA* gene in this pathogen. Two key silenced mutants, ΔVeA‐1 and ΔVeA‐2, were constructed to systematically investigate their biological phenotypes and regulatory mechanisms in conferring disease resistance against *P. heterophylla* .

**FIGURE 6:**
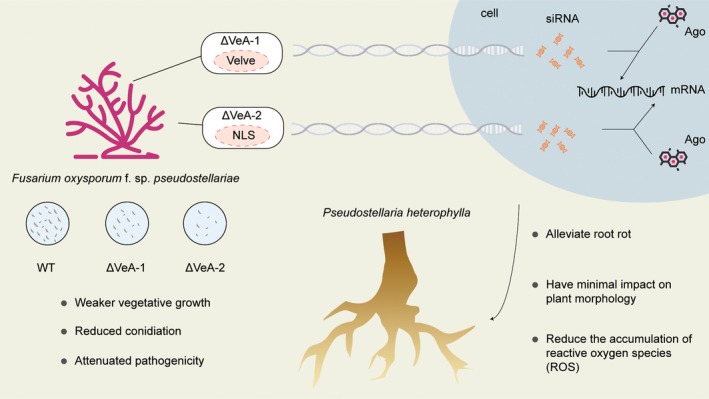
This schematic diagram illustrates the biological phenotypes and disease resistance mechanisms of the *VeA* silencing mutants (ΔVeA‐1 and ΔVeA‐2), which target the functional domains of the *VeA* gene (Velvet protein and nuclear localization signal, NLS) in *Fusarium oxysporum* f. sp. *pseudostellariae*. The left panel shows that, compared with the wild type (WT), the mutants exhibit impaired vegetative growth, reduced conidiation and attenuated pathogenicity. The right panel depicts their disease resistance effect on *Pseudostellaria heterophylla*, achieved through the intracellular pathway in which small interfering RNA (siRNA) and Argonaute (Ago) proteins act on messenger RNA (mRNA), thereby alleviating root rot, maintaining normal plant morphology and reducing reactive oxygen species (ROS) accumulation.

In Figure 6, we demonstrate the biological phenotypes and disease resistance mechanisms of the *VeA* silencing mutants (ΔVeA‐1 and ΔVeA‐2) in Fop. At the phenotypic level, compared with the WT strain, both ΔVeA‐1 and ΔVeA‐2 exhibit a distinct ‘triple attenuation’ phenotype. First, their vegetative growth is impaired, as evidenced by weakened hyphal expansion and reduced metabolic activity. Second, conidiation (spore production) is markedly decreased—because spores serve as the primary medium for pathogen transmission and infection, this reproductive inhibition directly reduces the potential for disease spread. Third, pathogenicity is substantially attenuated, with the infectivity of the mutants toward *P. heterophylla* significantly diminished, thereby establishing a biological foundation for disease resistance.

At the molecular level, the silenced strains markedly modify their pathogenicity by regulating the intracellular siRNA–Ago–mRNA pathway in Fop cells. Specifically, these mutant strains effectively suppress the development of root rot, allowing *P. heterophylla* to maintain normal root growth by preventing pathogen invasion and tissue destruction. Moreover, the silenced strains exert minimal influence on plant morphology, enabling *P. heterophylla* to sustain normal growth, development and physiological activity. In addition, they significantly reduce the accumulation of ROS in *P. heterophylla* , thereby preventing oxidative stress–induced secondary damage and maintaining intracellular homeostasis.

Taken together, this comparative analysis between WT and VeA‐deficient strains enabled us to construct an integrated mechanistic model encompassing fungal infection behaviour, host oxidative stress responses and immune‐related gene activation. This work provides a representative framework for dissecting the pathogenic mechanisms of filamentous fungi and their interactions with plant defence systems.

## 3 Discussion

**FIGURE 7:**
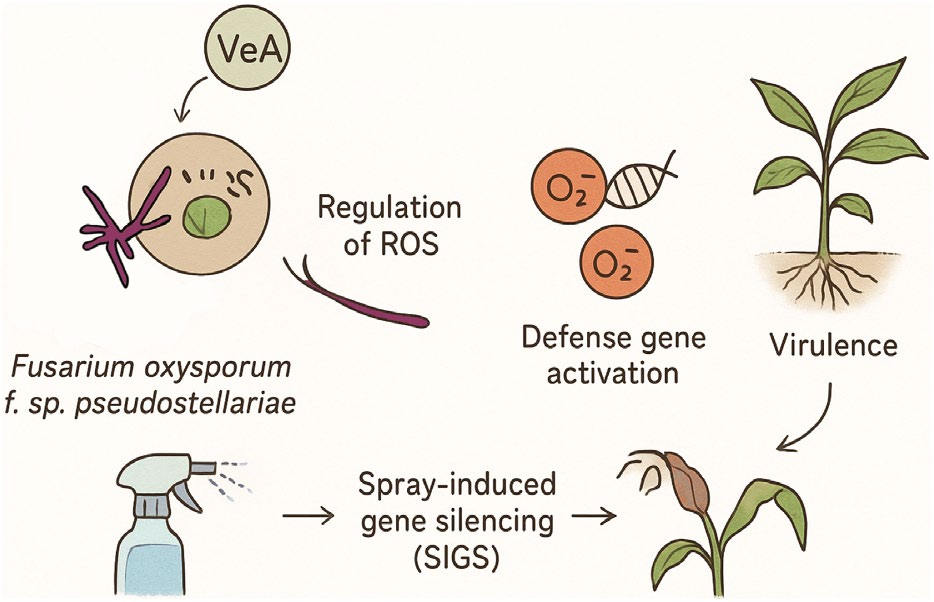
The pathogenesis and disease resistance mechanism of *Fusarium oxysporum* f. sp. *pseudostellariae* infection in *Pseudostellaria heterophylla* mediated by VeA regulation, reactive oxygen species (ROS)‐mediated defence gene activation and spray‐induced gene silencing (SIGS).

This study systematically investigated the function of the *VeA* gene in Fop, the host‐specialized pathogen responsible for root rot in *P. heterophylla* , using domain‐specific RNAi. By selectively silencing the conserved Velvet domain (ΔVeA‐1) and the nuclear localization signal (NLS) region (ΔVeA‐2), we dissected VeA's roles in fungal development, virulence and host response. Below, we interpret these findings in the context of current knowledge, highlighting key innovations, discussing limitations and proposing directions for future research (Figure 7).

Phylogenetic and structural analyses confirmed that Fop's VeA belongs to the canonical Velvet regulatory protein family. Notably, Fop VeA contains an N‐terminal Velvet domain (aa 1–110) responsible for protein–protein interactions (e.g., with VelB and LaeA) and a C‐terminal NLS (aa 347–357) essential for nuclear transport (Bayram et al. 2008; Stinnett et al. 2007).

RNAi experiments confirmed the functional importance of these domains. Silencing the NLS (ΔVeA‐2) reduced VeA transcript levels by ~70%, more effectively than silencing the Velvet domain (ΔVeA‐1, ~20%) and also resulted in stronger inhibition of colony growth and sporulation. These findings underscore the indispensable role of VeA nuclear localization in its regulatory activity (Strohdiek et al. 2025). The nuclear function of VeA in Fop may have undergone adaptive evolution to match the specific biology of *P. heterophylla* , expanding our understanding of Velvet proteins in host‐specific pathogenicity.

### 3.1 VeA Links Fungal Fitness and Virulence in the Fop– *P. heterophylla* System

*VeA* silencing significantly impaired Fop vegetative growth and reproductive fitness consistent with the conserved role of Velvet proteins in asexual development (Dhingra et al. 2012; Wang et al. 2019). Importantly, reduced fitness directly correlated with decreased virulence (López‐Berges et al. 2013). This correlation indicates that VeA enhances Fop infectivity by maintaining reproductive capacity—a critical determinant of pathogenicity for soil‐borne, spore‐dependent pathogens. Furthermore, the virulence defect of ΔVeA‐2 was markedly stronger than ΔVeA‐1, demonstrating that nuclear localization is essential not only for fungal growth but also for its function as a virulence factor.

### 3.2 VeA May Indirectly Modulate Host Oxidative Stress Responses and Immune Reprogramming

VeA also modulates Fop's ability to manipulate host defence, particularly oxidative stress and immune pathways.

#### 3.2.1 Oxidative Stress

Wild‐type Fop infection induced a strong oxidative burst in *P. heterophylla* , increasing antioxidant enzyme activities compared with uninfected controls, consistent with the role of ROS in plant–pathogen interactions, where excessive ROS promotes host cell death (Sharma et al. 2012; Huang et al. 2019). In contrast, ΔVeA strains induced significantly lower enzyme activity, indicating VeA is critical for Fop‐mediated destructive oxidative stress, likely contributing to root rot and tissue necrosis.

#### 3.2.2 Immune Pathways

Transcriptome analyses revealed that VeA triggers immune reprogramming in the host. Energy metabolism reprogramming: enrichment of amino sugar metabolism pathways supports ROS detoxification and defence protein synthesis (Tsuda and Somssich 2015). Pathogenesis‐related (PR) gene expression: *PR2*, *PR4* and *PR5* were 3–5 times more highly expressed than in controls and ΔVeA‐infected plants, contributing to fungal cell wall degradation (Dodds and Rathjen 2010). In ΔVeA infections, PR genes were significantly upregulated compared with the WT group, suggesting that VeA facilitates Fop in overcoming basal immunity through overactivation of host defences. This ‘immune exhaustion’ adds a new dimension to the cross‐species regulatory function of Velvet proteins, previously considered primarily regulators of fungal secondary metabolism (Weiberg et al. 2013; Adejor et al. 2025).

### 3.3 Future Directions

#### 3.3.1 Implications for Biological Control

VeA represents a promising target for controlling Fop‐induced root rot. Domain‐specific RNAi (e.g., targeting the NLS) can enable precise and efficient silencing. Because ΔVeA‐2 had a stronger inhibitory effect on virulence than ΔVeA‐1, NLS‐targeted approaches may be more effective. This strategy informs optimization of RNAi technologies, including SIGS, a non‐transgenic plant protection method gaining attention (Koch et al. 2016; Chauhan and Rajam 2022; Chen et al. 2025). Additionally, understanding VeA's modulation of host immunity can guide breeding of *P. heterophylla* genotypes with balanced ROS detoxification and enhanced PR gene expression, promoting stronger root rot tolerance.

#### 3.3.2 RNAi vs. Knockout

RNAi reduced *VeA* transcripts by 50%–70%, leaving residual activity; CRISPR‐Cas9 knockout mutants could more definitively reveal VeA's essential functions. However, the CRISPR‐Cas9 system cannot yet be widely applied under field conditions, which limits its practical utility and economic benefits. In contrast, RNAi technology offers a more feasible and flexible alternative. Through SIGS, specifically designed double‐stranded RNA (dsRNA) molecules can target and silence key virulence genes of pathogens, thereby achieving effective disease control. Notably, RNAi technology provides unmatched adaptability in targeting distinct conserved structural domains within pathogenic genes.

In the pSilent system, the designed target fragments are processed by the classical Dicer enzyme to generate siRNAs. These siRNAs subsequently guide the sequence‐specific degradation of the corresponding target mRNAs. As different target regions give rise to different siRNAs, the silencing efficiency may vary depending on the domain selected. In this study, the RNAi construct targeting the NLS domain exhibited a higher silencing efficiency than that targeting the Velvet domain. Quantitative expression analysis further confirmed a marked reduction in *VeA* transcript abundance. The precise molecular mechanisms underlying this process remain to be explored.

#### 3.3.3 Secondary Metabolites

VeA likely regulates secretion of effectors or toxins, which are critical for *Fusarium* virulence (Guo et al. 2014). Transcriptomic or metabolomic profiling of ΔVeA strains could identify these VeA‐dependent factors.

#### 3.3.4 Field Validation

Controlled laboratory conditions may not fully reflect agricultural environments; field trials are required to assess the efficacy of SIGS and resistant varieties.

## 4 Experimental Procedures

### 4.1 Plant and Fungal Materials and Culture Conditions

The *P. heterophylla* materials used in this study were collected from a commercial cultivation base in Zherong, Fujian, China. Fresh tissue culture seedlings maintained in the laboratory were rinsed thoroughly with tap water, air‐dried in a laminar‐flow hood, surface‐sterilized with 70% ethanol for 30 s, and subsequently washed four or five times with sterile distilled water (SDW). The disinfected seedlings were transferred onto sterile filter paper in Petri dishes for further processing. Under aseptic conditions, stem tips approximately 1 cm in length were excised and inoculated on Murashige and Skoog (MS) medium supplemented with 100 μL/L 1‐naphthaleneacetic acid (NAA) (pH 5.8 ± 0.1). Cultures were incubated at 25°C ± 1°C with a 16‐h light photoperiod.

The *F. oxysporum* strain used in this study was originally isolated from the rhizosphere of *P. heterophylla* plants in Zherong, Fujian, China. Fungal plugs of uniform size were obtained from potato dextrose agar (PDA) plates of the laboratory‐preserved Fop strain using a sterile hole punch and transferred to fresh PDA. Cultures were incubated in darkness at 28°C in a constant‐temperature incubator for the entire growth period.

### 4.2 Extraction of Fop Genomic DNA

Genomic DNA of the WT Fop was extracted using a commercial fungal genomic DNA extraction kit (Solarbio). The strain was cultured on PDA at 28°C in darkness for 7 days. Mycelia were harvested and ground into a fine powder in liquid nitrogen, followed by genomic DNA extraction according to the manufacturer's instructions. DNA quality and concentration were assessed using 1% agarose gel electrophoresis and a NanoDrop spectrophotometer. Verified DNA samples were stored at −20°C.

### 4.3 PCR Amplification and Sequencing of the *VeA* Gene

Specific primers were designed using Primer Premier 5.0 software based on the *VeA* gene sequence of *Fusarium* spp. deposited in GenBank (accession number XM_018390838.1). The forward primer was VeA‐F (5′‐ATGGCCTATCTCGACCGTCC‐3′) and the reverse primer was VeA‐R (5′‐TCATAATACCGGTTGAACTGGACGG‐3′). PCR amplification was performed using Fop genomic DNA as the template. Each 50 μL reaction contained 25 μL of 2× Lab SuperHF PCR Master Mix (Lanbode), 2 μL of each primer (10 μM), 2 μL of template DNA and 19 μL of sterile water. The amplification programme consisted of an initial denaturation at 95°C for 5 min; followed by 34 cycles of denaturation at 98°C for 30 s, annealing at 57°C for 30 s and extension at 72°C for 1 min; with a final extension at 72°C for 5 min. PCR products were analysed by 1% agarose gel electrophoresis, and the target band was excised, purified, and submitted to Sangon Biotech (Shanghai, China) for sequencing.

### 4.4 VeA Sequence Analysis

The open reading frame (ORF) of the *VeA* gene obtained from sequencing was analysed using DNAMAN software, and the corresponding amino acid sequence was deduced. Homologous proteins were identified through BLASTp searches in the NCBI database. A phylogenetic tree was constructed using the neighbour‐joining (NJ) method implemented in MEGA 11, with 1000 bootstrap replications to assess branch confidence. Conserved protein domains were predicted using the InterPro database. The NLS of VeA was predicted using two complementary online tools, DeepLoc‐2.0 (DTU Health Tech) and cNLS Mapper (Figure S2).

### 4.5 RNAi Silencing Vector Construction

Based on the conserved domain prediction results of the VeA protein, specific primer pairs targeting the Velvet domain and the NLS region were designed for RNA silencing. Homologous arm sequences corresponding to the pSilent vector were incorporated at both primer ends. Using the amplified *VeA* gene as the template, the VeA‐1 and VeA‐2 fragments were generated by PCR under the same reaction conditions described above. The purified VeA‐1 and VeA‐2 fragments, along with the pSilent‐1 vector, were digested with XhoI and HindIII. Following agarose gel electrophoresis and recovery, the recombinant silencing plasmids pSilent‐VeA‐1 and pSilent‐VeA‐2 were assembled using the Hieff Clone Universal One‐Step Cloning Kit (Yeasen Biotech) at 50°C for 15 min. The resulting constructs were transformed into *Escherichia coli* DH5α competent cells, and transformants were screened by colony PCR after overnight incubation. Positive clones were verified by sequencing at Sangon Biotech (Shanghai, China).

### 4.6 Preparation and Transformation of Fop Protoplasts

The Fop strain was cultured on PDA at 28°C in darkness for 5 days. The cultures were then chilled at 4°C for 30 min. Fresh mycelia were harvested into centrifuge tubes and washed with 1 mL of precooled phosphate‐buffered saline (PBS) (4°C). Subsequently, 1 mL of filter‐sterilized enzymolysis solution (cellulase, lywallzyme, chitinase and β‐glucanase; all precooled to 4°C) was added. The suspension was incubated at 37°C and 200 rpm for 4 h to generate fresh protoplasts. After washing with PBS, residual mycelial fragments were removed using four layers of sterile lens paper, and the purified protoplasts were stored at 4°C for transformation.

For transformation, 1 mL of protoplast suspension was mixed with 3–5 μg of plasmid DNA, followed by the addition of 300 μL of SPTC solution (1 M sorbitol, 10 mM Tris–HCl, 10 mM CaCl_2_ and 40% polyethylene glycol [PEG] 4000). The mixture was gently blended and incubated on ice in darkness for 30 min. An additional 700 μL of SPTC solution was then added and mixed, followed by incubation at room temperature in darkness for 20 min. The transformed protoplasts were transferred to regeneration medium (PDA with 1.2 M sorbitol, 10 mM CaCl_2_) and incubated upside down at 28°C in darkness for 10–12 h. A layer of selection medium (PDA with 1.2 M sorbitol, 10 mM CaCl_2_ and 1% hygromycin) was overlaid, and plates were further incubated at 28°C in darkness for 4–5 days. Emerging colonies were transferred to PDA supplemented with hygromycin for purification, yielding candidate silencing transformants ΔVeA‐1 and ΔVeA‐2 (Meyer 2008).

Total RNA was extracted from ΔVeA‐1 and ΔVeA‐2 strains, followed by reverse transcription. Positive transformants were confirmed by PCR amplification of the *hyg* resistance gene carried on the pSilent vector.

### 4.7 Gene Expression Analysis

Total RNA was extracted from the WT, ΔVeA‐1 and ΔVeA‐2 strains, as well as from *P. heterophylla* samples from the CK, WT, ΔVeA‐1 and ΔVeA‐2 treatment groups. First‐strand cDNA was synthesized using the Evo Super M‐MLV Reverse Transcriptase Polymerase II kit (Accurate Biology) according to the manufacturer's protocol. Real‐time qPCR was performed with Taq‐HS Probe qPCR Premix (Lablead) on a LightCycler 96 system (Roche) to quantify relative gene expression. *β‐actin* served as the internal reference gene for both Fop and *P. heterophylla* . Each 10 μL qPCR contained 5 μL Taq‐HS Premix, 0.4 μL of each primer (10 μM), 1 μL of cDNA and 3.2 μL of nuclease‐free water. Thermal cycling conditions were as follows: 95°C for 30 s, followed by 40 cycles of 95°C for 5 s and 60°C for 30 s. A melting curve was generated using the instrument's default settings. Relative gene expression was calculated using the 2^−ΔΔCt^ method. All samples were analysed with three biological replicates and four technical replicates.

### 4.8 Determination of Colony Morphology and Growth Rate of Fop

The WT, ΔVeA‐1 and ΔVeA‐2 strains were individually inoculated at the centre of PDA plates and incubated at 28°C for 7 days. Colony diameters were measured daily for five consecutive days using the cross method, and growth curves were generated to calculate growth rates (cm/day). Three biological replicates were included for each strain. On Day 7, colony morphology, including pigmentation, mycelial density and aerial hyphae characteristics, was examined and documented.

### 4.9 Determination of Spore Production of Fop

The WT, ΔVeA‐1 and ΔVeA‐2 strains were inoculated into potato dextrose broth (PDB) and incubated at 28°C in darkness for 3 days. A 10 mL aliquot of the resulting spore suspension was collected and filtered through four layers of sterile lens paper to remove mycelial debris. Then, 10 μL of the filtered suspension was loaded onto a haemocytometer, and spores were counted under a light microscope to calculate spore yield (spores/cm^2^ of colony area). Each strain included three biological replicates.

### 4.10 Calculation of Disease Index of *P. heterophylla*

The WT, ΔVeA‐1 and ΔVeA‐2 strains were inoculated into PDB and cultured with shaking at 28°C, 180 rpm for 3 days to obtain spore suspensions, which were adjusted to 10^6^ spores/mL. Uniformly growing *P. heterophylla* seedlings were selected and inoculated by rhizosphere drenching with 10 μL of spore suspension per plant. Sterile water was used as the control (CK). Following inoculation, the seedlings were maintained in a tissue culture room for 14 days. Plant growth status was recorded on Days 7 and 14. Disease index was calculated as follows:

$$\mathrm{DI}=\frac{\sum \left(\text{individual score}\right)}{\text{total number of plants}\times 100}\times 100$$

The grading criteria are described as Table 1.

**TABLE 1:** Disease grading criteria for Fusarium wilt of *Pseudostellaria heterophylla*.

Disease grade	Symptom description	Score
0	Root system healthy; no discoloration or rot observed; shoots and leaves remain green and turgid.	0
1	Slight browning of fine roots; no apparent rot; leaves show no or very mild wilting.	1–20
2	Partial root rot affecting ≤ 30% of root area; moderate browning of main roots; slight leaf wilting.	20–40
3	Moderate root rot affecting 30%–60% of root area; obvious cortical decay; leaves exhibit noticeable wilting.	40–60
4	Severe root rot affecting > 60% of root area; main roots decayed and softened, leaves exhibit noticeable wilting.	60–80
5	Root system completely rotted; plant nearly or completely dead.	80–100

### 4.11 Determination of Antioxidant Enzyme Activity of *P. heterophylla*

Fourteen days after inoculation, root tissues of *P. heterophylla* from the CK, WT, ΔVeA‐1 and ΔVeA‐2 groups were collected and ground to a fine powder in liquid nitrogen. Approximately 0.1 g of cleaned tissue was placed in a prechilled mortar, and 5 mL of ice‐cold 50 mM phosphate buffer (pH 7.8) was added. Samples were homogenized on ice, transferred to centrifuge tubes and centrifuged at 12,000 *g* for 20 min at 4°C. The resulting supernatant was used for antioxidant enzyme activity assays. POD, CAT and SOD activities were quantified as follows: POD activity was determined by the guaiacol oxidation method, where one unit (U·g^−1^ FW) corresponds to an increase of 0.01 in absorbance at 470 nm per minute. CAT activity was measured by UV spectrophotometry, with one unit defined as a decrease of 0.01 in absorbance at 240 nm per minute. SOD activity was assessed using the nitroblue tetrazolium (NBT) photoreduction method, with one unit defined as the amount of enzyme required to inhibit 50% of NBT photoreduction. Six replicates were included for each treatment.

### 4.12 Screening and Functional Enrichment Analysis of Differentially Expressed Genes (

*Pseudostellaria heterophylla* root samples from the CK and WT treatment groups were subjected to transcriptome sequencing on the Illumina NovaSeq 6000 platform. After quality filtering, clean reads were aligned to the *P. heterophylla* reference genome. Differentially expressed genes (DEGs) were identified using DESeq2 with the thresholds log_2_FC > |1| and *p*_adj_ < 0.05. The top 50 DEGs were selected for hierarchical clustering to visualize expression patterns. Gene Ontology (GO) biological process and Kyoto Encyclopedia of Genes and Genomes (KEGG) pathway enrichment analyses were performed using the ClusterProfiler R package, with significantly enriched terms defined at *p*_adj_ < 0.05.

### 4.13 Statistical Analysis of Data

All experimental data were analysed using SPSS v. 26.0. Differences between two groups were assessed using independent‐sample *t*‐tests, while comparisons among multiple groups were evaluated by ANOVA followed by Duncan's multiple range test to determine statistical significance. Graphs were generated using GraphPad Prism v. 9.0. Statistical significance was defined as follows: *p < 0.05, **p < 0.01, ***p < 0.001 and ****p < 0.0001.

### 4.14 qPCR for *VeA* Gene Copy Number Determination

The genomic copy number of *VeA* in Fop was determined by absolute qPCR using the single‐copy gene *tubulin* as a reference. Genomic DNA was extracted from mycelia, and gene‐specific primers were designed for *VeA* and *tubulin* (Table S1). Standard curves were generated using 10‐fold serial dilutions of purified PCR products with known copy numbers. qPCR was performed with SYBR Green chemistry on a Bio‐Rad CFX96 system. Three biological replicates with three technical replicates each were analyzed. The *VeA* copy number was calculated based on the standard curve and normalized to *tubulin* to determine copies per haploid genome.

### 4.15 Absolute Quantification of Fungal Biomass by qPCR

Fungal biomass in infected *P. heterophylla* roots was quantified by qPCR. Total genomic DNA was extracted from root tissues using a plant DNA extraction kit following the manufacturer's protocol. Fop‐specific primers targeting a genomic region of Fop were used for fungal DNA detection (Table S1). Standard curves were generated using 10‐fold serial dilutions of purified Fop genomic DNA with known concentrations. qPCR was performed using SYBR Green chemistry on a Bio‐Rad CFX96 Real‐Time PCR system. Three biological replicates with three technical replicates each were analysed. Fungal biomass was calculated based on the standard curve and expressed as the absolute amount of fungal DNA per unit total root DNA.

## Author Contributions

**Yanyang Jiao:** resources. **Zhen Fang:** conceptualization, methodology, investigation, writing – original draft. **Wenxiong Lin:** writing – review and editing, funding acquisition, project administration, supervision. **Chenjing Zhang:** resources. **Shengen Zhong:** visualization. **Ludi Yang:** validation, data curation. **Yuping Li:** visualization, formal analysis. **Jianing Guo:** visualization. **Quancheng Sun:** data curation, investigation.

## Conflicts of Interest

The authors declare no conflicts of interest.

## Supporting information


**Figure S1:** Multiple sequence alignment of Fop‐VeA.

**Figure S2:** Bioinformatic analysis of conserved domains and nuclear localization signals in the *Fusarium oxysporum* f. sp. *pseudostellariae* VeA protein.

**Figure S3:** Standard curves for absolute quantitative real‐time PCR (qPCR) analysis of *VeA* gene copy number in *Fusarium oxysporum* f. sp. *pseudostellariae*.

**Figure S4:** Validation of the pSilent recombinant vector and amplification of *VeA*.

**Figure S5:** Relative expression of RNAi pathway genes in *Fusarium oxysporum* f. sp. *pseudostellariae*.

**Table S1:** All primers used in this study.

## Data Availability

The main data supporting the findings of this study are available within the article and its Supporting Information. Extra data are available from the corresponding author upon reasonable request.
