# Supplementary figures and images for: Domain‐Targeted RNAi of VeA Reveals Its Essential Role in the Fusarium oxysporum–Pseudostellaria heterophylla Interaction
Source: Mol Plant Pathol. 2026 Apr 24;27(4):e70257. doi: 10.1111/mpp.70257 (PMC13109616; doi:10.1111/mpp.70257)

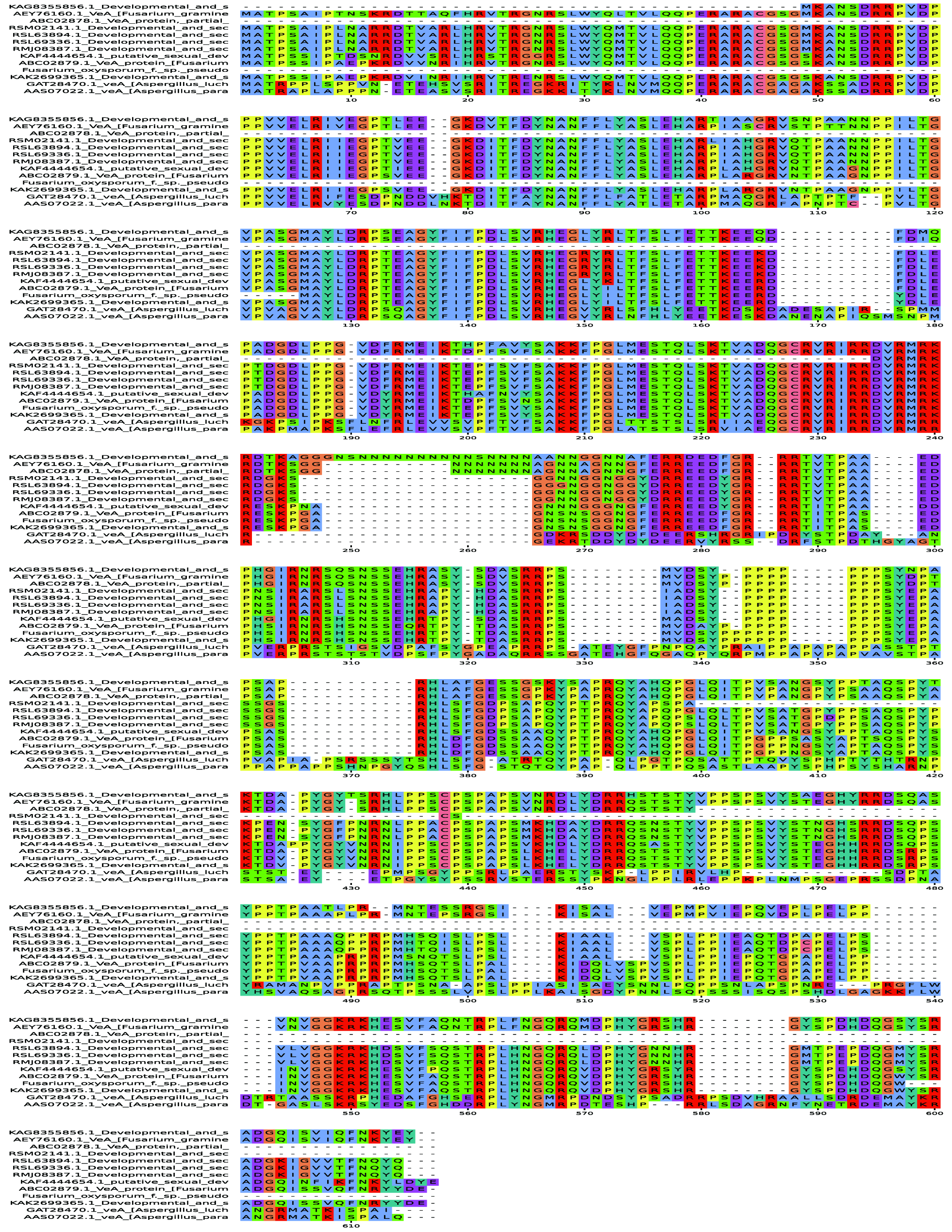

Supplement: Supplementary file 1 — Figure S1: Multiple sequence alignment of Fop‐VeA. [file MPP-27-e70257-s004.tif]

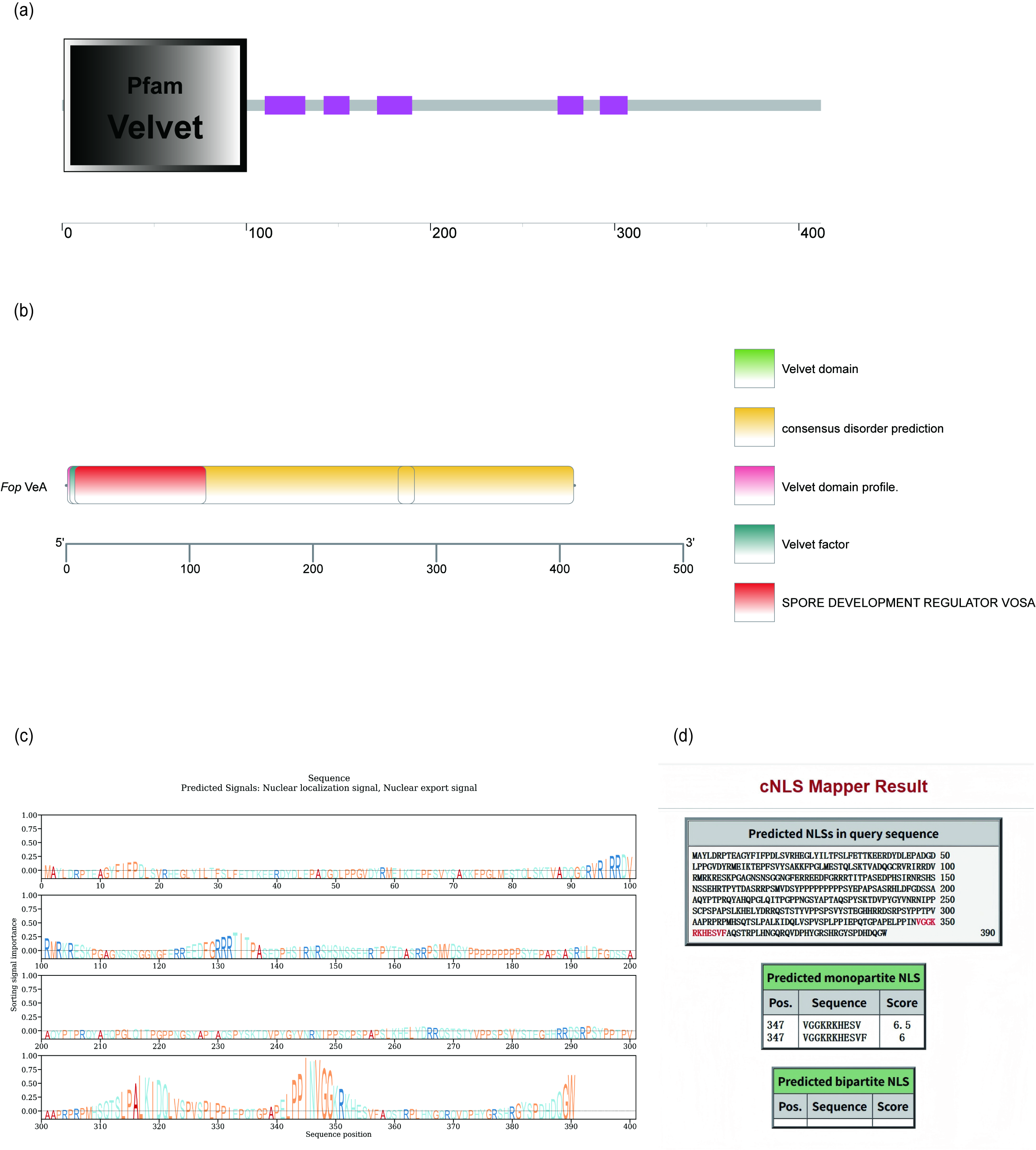

Supplement: Supplementary file 2 — Figure S2: Bioinformatic analysis of conserved domains and nuclear localization signals in the Fusarium oxysporum f. sp. pseudostellariae VeA protein. [file MPP-27-e70257-s001.tif]

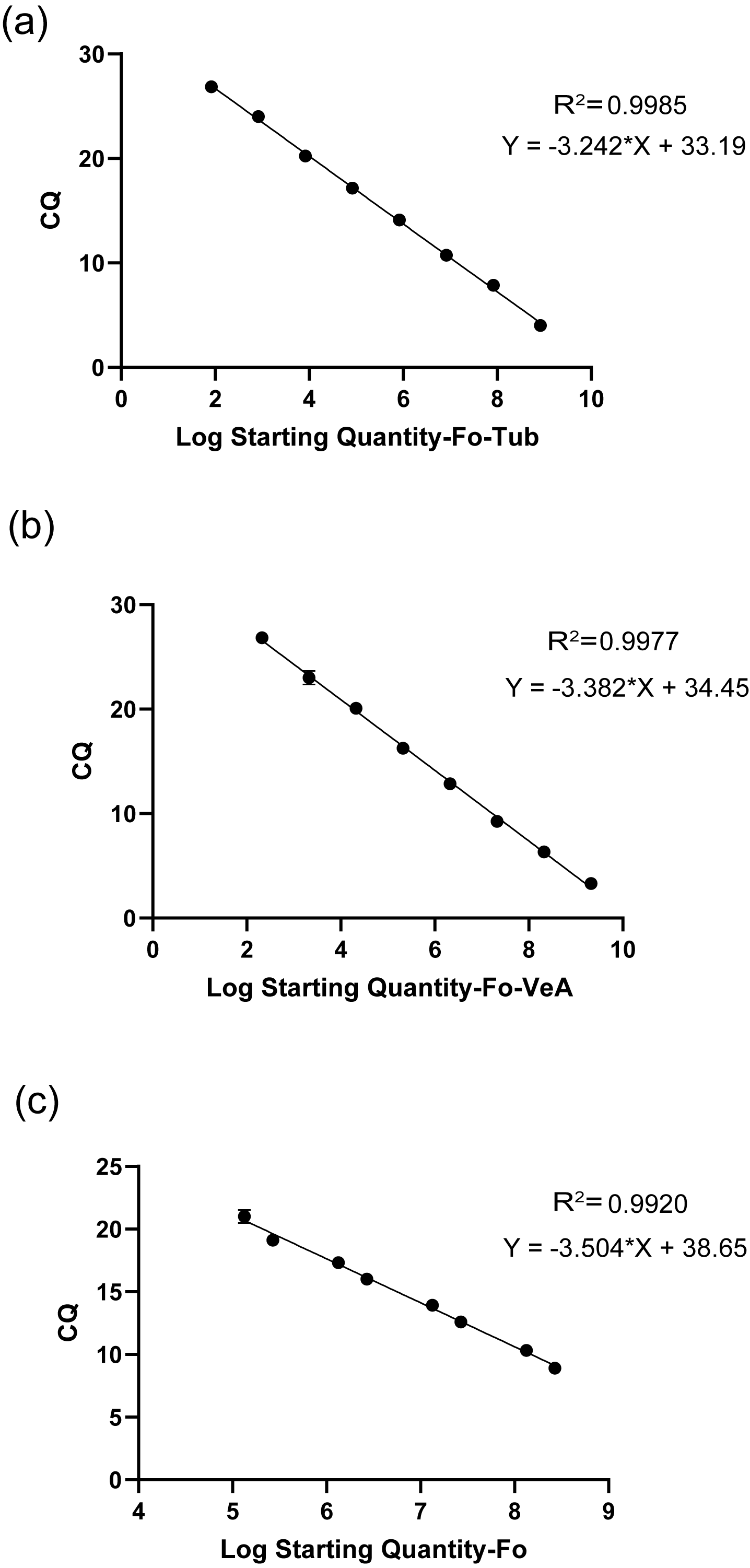

Supplement: Supplementary file 3 — Figure S3: Standard curves for absolute quantitative real‐time PCR (qPCR) analysis of VeA gene copy number in Fusarium oxysporum f. sp. pseudostellariae. [file MPP-27-e70257-s006.tif]

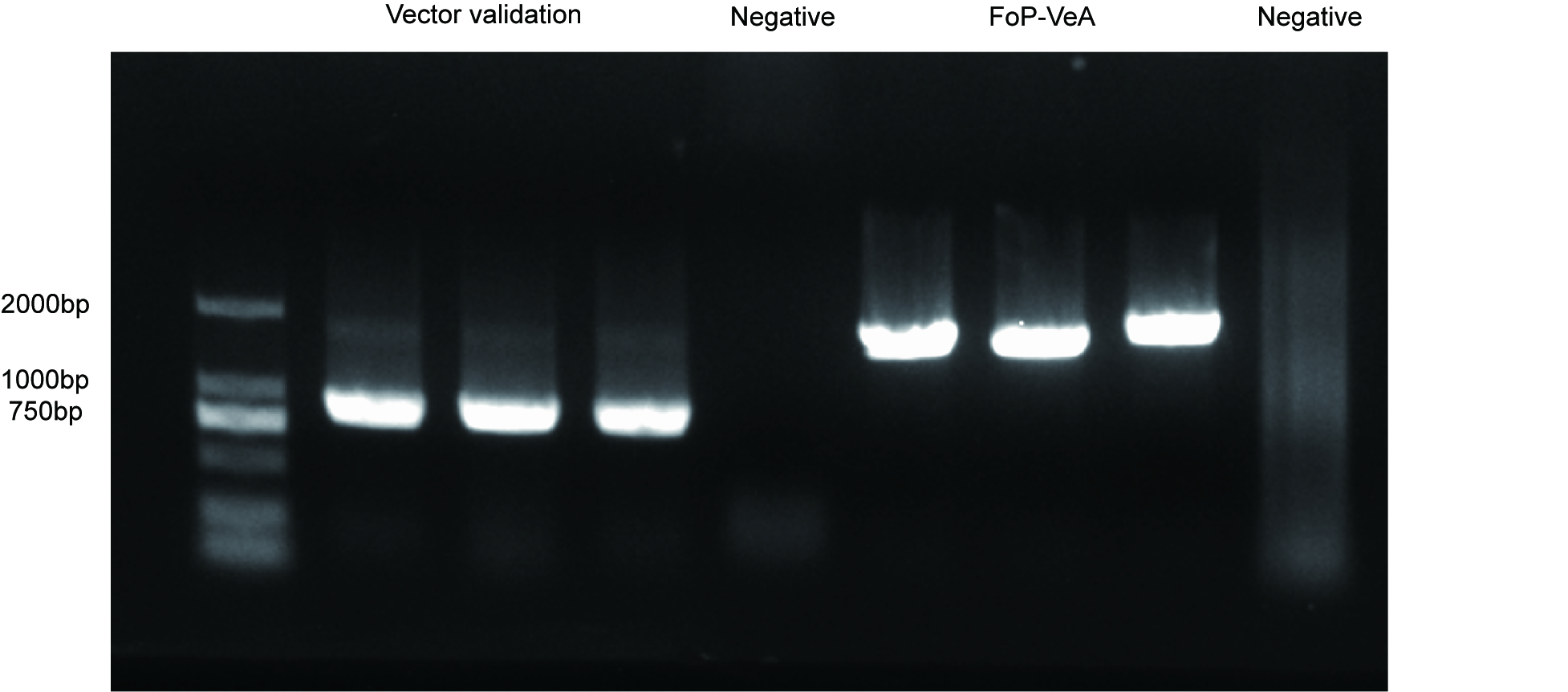

Supplement: Supplementary file 4 — Figure S4: Validation of the pSilent recombinant vector and amplification of VeA. [file MPP-27-e70257-s003.tif]

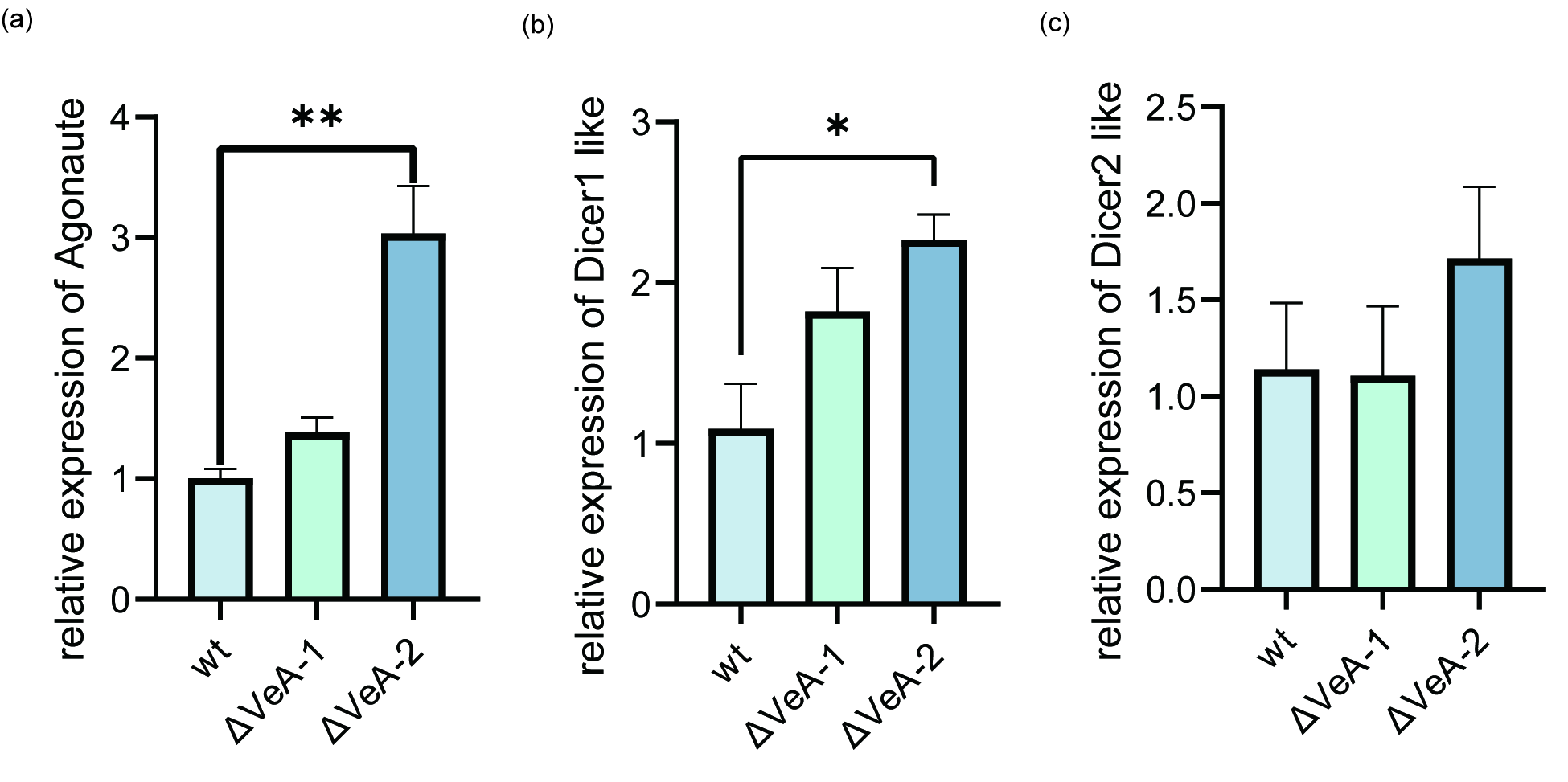

Supplement: Supplementary file 5 — Figure S5: Relative expression of RNAi pathway genes in Fusarium oxysporum f. sp. pseudostellariae. [file MPP-27-e70257-s005.tif]
