# Supplementary material for: Domain‐Targeted RNAi of VeA Reveals Its Essential Role in the Fusarium oxysporum–Pseudostellaria heterophylla Interaction
Source: Mol Plant Pathol. 2026 Apr 24;27(4):e70257. doi: 10.1111/mpp.70257 (PMC13109616; doi:10.1111/mpp.70257)
Supplement: Supplementary file 6 — Table S1: All primers used in this study. [file MPP-27-e70257-s002.docx]

**Table. S1: All primers used in this study**

| **Primer name** | **Primer sequence(5’-3’)** | **Application** |
| --- | --- | --- |
| Fo-VeA-qF | CTCCTTCTGCATCGCGTCAC | qPCR |
| Fo-VeA-qR | GCATAAGAACCATTGGGCGGAC | qPCR |
| Fo-800a-F | TTCCTGCCGAGCCAAAGC | PCR |
| Fo-800a-R | AAACCGTTTCCACCACTGTTGC | PCR |
| Fo-800b-F | CGAACGTCGGGAAGAGGAC | PCR |
| Fo-800b-R | TTACCAGCCTTGGTCATGATCGG | PCR |
| Ago-qF | CTGCAGATCGGTCAAGATCCG | qpcr |
| Ago-qR | GTTCGGCTGTACCTTGTCCAAA | qpcr |
| dlc1-qF | AAGATACCTTCGACGGGCG | qpcr |
| dlc1-qR | CGATCGCCAACCTTCTGTACT | qpcr |
| dlc2-qF | AGATGACCAACGAGAATTGGCTC | qpcr |
| dlc2-qR | CAGGTGTTGTCGAGCATTGTCG | qpcr |
| 11273-F | ATGGCTACACCATCCTCGATTCC | PCR |
| 11273-R | CTACTCGTCATAATACCGGTTGAACTGG | PCR |
| Ph-actin-qF1 | CTGTATTTACGCTCAGGTGG | qpcr |
| Ph-actin-qR1 | CATTGTGCTCAGTGGTGG | qpcr |
| Ph-PR5-qF | TGTGAGGCGTTTGGGGATTC | qpcr |
| Ph-PR5-qR | CGTAGCTATACGCTCTTGGACACG | qpcr |
| Ph-PR2-qF | TCAACAGTATGGTTGGACTGCCT | qpcr |
| Ph-PR2-qR | TCCACTATTCTCACTGTTGTTTGTGCT | qpcr |
| Ph-PR4-qF | GGTTACGGTGAGAACATTGCGAT | qpcr |
| Ph-PR4-qR | CCACACATTTGGCCTTCCGTACAA | qpcr |
| Fo_actin_F | ACCTAGGCGAGATGGACCTT | qpcr |
| Fo_actin_R | TCCGATGAGTTCACGCTGAG | qpcr |
| pS-det-F | cccaagcatcgataccgtcg | det |
| pS-det-R | tagggcgaattgggatcctct | det |
| pS-Hyg-F | atgaaaaagcctgaactcaccgc | det |
| pS-Hyg-R | ttcctttgccctcggacga | det |
| Fo-tub-F | CAGATTTATCTCATCGGCGCT | qPCR |
| Fo-tub-R | TGACCTTATGTGAGCCCACT | qPCR |
| Fo-tub-qF | TGCACTGTCTCTCACGGTTCT | qPCR |
| Fo-tub-qR | GGACCGACAGCGTGGCATTATA | qPCR |
| Fo-its-F | CTTGGTCATTTAGAGGAAGTAA | qPCR |
| Fo-its-R | CGAATTAACGCGAGTCCCAA | qPCR |
